# Expression of COX-1 and COX-2 in the endometrium of cyclic, pregnant and in a model of pseudopregnant rats and their regulation by sex steroids

**DOI:** 10.1186/1477-7827-8-103

**Published:** 2010-08-24

**Authors:** Isabelle St-Louis, Mohan Singh, Kevin Brasseur, Valérie Leblanc, Sophie Parent, Eric Asselin

**Affiliations:** 1 Research group in molecular oncology and endocrinology, Department of chemistry-biology, Université du Québec à Trois-Rivières, C.P. 500, Trois-Rivières, Québec, G9A 5H7, Canada

## Abstract

**Background:**

Cyclooxygenases (COXs) are the rate limiting enzymes in the process of prostaglandins (PGs) synthesis, which are critical regulators of a number of reproductive processes, including ovulation, implantation, decidualization and parturition. The aim of the present study was to investigate the expression and regulation of COX-1 and COX-2 and levels of prostaglandins during rat pregnancy, in a model of pseudopregnancy and estrous cycle.

**Methods:**

Uteri were collected from the cyclic rats on each day of estrous cycle, after every two days for pregnant (days 2 to 22) and pseudopregnant rats (days 1 to 9). In vitro primary endometrial stromal cells were cultured in the presence of steroid hormones and their respective inhibitors for the possible modulation of COX-1 and COX-2. Endometrial protein extracts were used for western blot analysis and tissue sections were prepared for protein localization using immunofluorescence. Measurements of PGF2alpha and PGE2 metabolites in serum were performed by enzyme immunoassay (EIA).

**Results:**

COX-1 expression was found to be elevated during implantation and parturition, however, the levels of COX-1 decreased during decidualization periods. COX-2 was detected during early pregnancy from day 2 to 5, increased during decidual regression, and was also expressed at the time of parturition. COX-2 protein expression was found to be increased at estrus phase in cyclic rats. Both enzymes were found to be modulated in the endometrium of pseudopregnant rats, suggesting that they are regulated by 17beta-estradiol and progesterone. A significant increase in PGE2 metabolite levels was observed on day 10, 12 and 14 of pregnancy. However, an increase in PGF2alpha metabolite levels was observed only on day 14. The concentration of both these metabolites changed during pseudopregnancy and maximum levels were observed at day 7. Significant increase in PGE2 metabolite was observed at proestrus phase, on the other hand, PGF2alpha metabolite was significantly increased at proestrus and metestrus phase. COX-2 protein was regulated by 17beta-estradiol in cultured endometrial stromal cells which was blocked in the presence of ICI-182,780.

**Conclusions:**

Taken together, these results suggest that COX-1 and COX-2 could be differentially regulated by steroid hormones and might be the key factors involved in embryo implantation, decidualization, decidua basalis regression and parturition in rats.

## Background

Cyclooxygenase (COX), the rate-limiting enzyme in prostaglandin (PG) biosynthesis exists in two isoforms, COX-1 and COX-2. These enzymes catalyze the conversion of arachidonic acid into prostaglandin G2, which are further peroxidised to prostaglandins PGH2 [1]. COX-1 is known to be a constitutive enzyme expressed in most tissues [2], whereas expression of COX-2 can be induced by cytokines/growth factors or inflammatory stimuli. Previous studies suggest that COX-1 plays a crucial role during parturition [3], and COX-2 is important during ovulation, fertilization, implantation and decidualization [4]. Further, this fact is well supported by gene knockout studies; COX-1-deficient female mice are fertile with specific parturition defects, whereas COX-2-deficient females are infertile with abnormalities in ovulation, fertilization, implantation and decidualization [4,5]. The processes of ovulation and implantation are considered analogous to 'proinflammatory' responses; therefore, the involvement of PGs in these processes has long been speculated.

Prostaglandins are lipid mediators that play an important role in reproduction and maintainance of pregnancy [6,7]. PGE synthase (PGES) is a terminal prostanoid synthase and can enzymatically convert the cyclooxygenase product PGH2 to PGE2 which can further bind to and activate a set of functionally distinct cell surface receptors - EP1, EP2, EP3 and EP4 [8]. PGE2 and PGI2 are suspected to be implicated in the increase of vascular permeability during implantation and are known to be essential for decidualization [9]. Among various prostaglandins, PGF2α has also been considered as the primary candidate present during pregnancy, where PGF2α plays a crucial role in myometrium during parturition by increasing the oxytocin-induced contractions [10]. PGF2α is synthesized by PGF synthase and acts through FP receptor.

To date, the regulation and role of each COX enzyme and prostaglandin metabolite during pregnancy and estrous cycle in rat uterus have not been fully understood. Previously, we have shown that sex steroids regulate the expression of prostaglandin D synthase (PGDS) and prostacyclin synthase (PGIS) during pregnancy [11]. Therefore, the rationale of the present study is that if sex steroids could regulate the expression of PGDS and PGIS during pregnancy. Hence these steroids might play a pivotal role in the regulation of COX-1 and COX-2 which are the first and rate limiting enzymes for prostaglandin synthesis. In this study we investigated the endometrial expression of COX-1 and COX-2 and levels of prostaglandins metabolites during rat pregnancy, in a model of pseudopregnancy and during the estrous cycle.

## Methods

### Reagents

COX-1 (murine) polyclonal antibody (Cat. No.160109), and COX-2 (murine) affinity purified polyclonal antibody (Cat.No. 160126) were purchased from Cayman Chemicals (Ann Arbor, MI). Prostaglandin F2 metabolite 13,14-dihydro-15-keto PGF2α and prostaglandin E2 metabolite 11 beta-PGF2 were purchased from Cayman Chemicals (Ann Arbor, MI). Vectastain ABC Kit for rabbit IgG and Vector Nova Red were purchased from Vector Laboratories Inc. (Burlingame, CA, USA). 17β-estradiol (E2) and progesterone were purchased from Laboratoire Mat (QC, Canada).

### Animals

Sprague-Dawley female rats, 200-225 g, were obtained from Charles River Laboratories Canada. Animals were maintained on standard chow and water, which were available *ad libitum*, in animal facilities illuminated between 6:00 h and 20:00 h. All procedures were performed in accordance with guidelines of the Canadian Council on Animal Care for the handling and training of laboratory animals and the Good Health and Animal Care Committee of the Université du Québec à Trois-Rivières. Stages of the estrous cycle were confirmed by vaginal smears. Rats with three regular cycles of 4 days were used in these experiments and killed at various stages of the estrous cycle (diestrus, proestrus, estrus and metestrus). Also, male and female mice were mated overnight and pregnancy was confirmed by vaginal smears and/or the presence of a vaginal plug (day 1). Rats were killed on day 2, 4, 5, 6, 8, 10, 12, 14, 16, 18 and 20 of pregnancy at 10:00 h in the morning and at 18:00 h for days 5.5 and 6.5. Three to four different rats were used for each time of pregnancy. Uteri were collected and fixed for immunofluorescence staining (IF). Endometrial protein extracts were collected for western blot analysis.

### Pseudopregnancy model

A total of 3 rats per day of pseudopregnancy were ovariectomized and then allowed to recover from surgery for a minimum of 10 days. They were pre-treated with physiological doses of estradiol (1,3,5(10)-Estratriene-3,17β-diol, Sigma-aldrich) and progesterone (Laboratoire Mat, PQ) to induce decidualization as described previously (19): 1) 0.2 ug estradiol injection per day for three days (in the morning, day -2,-1 and 0); 2) On the third day (day 0 of pseudopregnancy), another injection in the afternoon of estradiol (0.2 μg) and progesterone (1 mg) was performed; 3) No treatment for 2 days (day 1 and 2 of pseudopregnancy); 4) Injections of estradiol (0.1 μg) and progesterone (4 mg) for three days (day 3, 4 and 5 of pseudopregnancy); 5) Another injection of estradiol (0.1 μg) in the afternoon on day 7 (day 4 of pseudopregnancy); 6) Rats were killed on day 1,3,5,7 and 9 of pseudopregnancy. Uteri were collected and fixed for immunofluorescence (IF) staining. Endometrial protein extracts were collected for western blot analysis.

### Endometrial stromal cell isolation, primary culture and *in vitro *decidualization

Uteri were removed from the model of pseudopregnant rats at day 5 using the same sex steroids treatment as described above. Horns were taken and immersed in HBSS solution containing HEPES (20 mM), penicillin (100 units/ml), streptomycin (100 μg/ml) and fungizone (1,25 μl/ml) (Invitrogen, ON, Canada). Further processing was performed in a sterile environment. The uterine horns were transferred into a sterile petri plate containing HBSS, slit longitudinally and immersed in trypsin type I solution (0.3%) (Roche Diagnostics, QC, Canada) in HBSS and agitated for 60 minutes at room temperature. Uterine horns were then vortexed at maximum for 5 sec and supernatant containing epithelial cells were discarded. Uterine horns were washed three times with 2.5 ml of HBSS and immersed in a HBSS solution containing trypsin type I (0.03%), DNAse I (0.016%) and collagenase type II (0.064%) for 15 minutes at 37°C in a water bath. Uterine horns were then vortexed again at maximum for 5 sec. The supernatant containing stromal cells was transferred into a sterile falcon tube containing 150 μl of FBS D.C. (Dextran-Charcoal extracted). Uterine horns were washed twice with 2.5 ml of HBSS and the supernatant was mixed with stromal cells. Uterine horns were discarded and stromal cells were centrifuged at 1000 g for 5 minutes. Cells were washed twice with HBSS and centrifuged. The supernatant was discarded and cells were diluted with DMEM-F12 (pH 7.1) (Invitrogen, ON, Canada) containing 2.438 g/L NaHCO3, 10% FBS D.C. and gentamycine 50 μg/ml. Cells were incubated at 37°C in the presence of 5% CO_2_. Cells were plated in 6-well plates (Corning plates) at a density of 50% (4 × 10^5 ^cells per well). The medium was changed two hours after the first incubation in order to eliminate epithelial cell contamination from stromal cell cultures. The purity of stromal cells was more than 97%: cell culture contamination with epithelial cells was evaluated by cellular morphology and immunofluorescence using a Keratin 8/18 antibody. Confluent cells (~90%) cells were then stimulated with steroids (estradiol and progesterone) in the presence and absence of respective blockers (ICI-182,780 and RU-486). The cells were pre-treated with ICI-182,780 and RU-486 at concentration of 1 μM for 1 hr, followed by the treatment of estradiol and progesterone at concentration of 100 nM for 48 h.

### Immunofluorescence staining

The uterus was fixed in 4% paraformaldehyde solution and embedded in paraffin. Tissue sections of 7 μm thickness were mounted on polylysine-coated slides. The slides were deparaffinized by heating at 60°C for 30 min followed by two washes in NeoClear solvent (VWR Canlab, Mississauga, Canada) and progressively hydrated with successive washes at room temperature in 100% ethanol, 95% ethanol in PBS, 70% ethanol in PBS, and followed by PBS. After permeabilization for 6 min in boiling citrate solution (0.1% sodium citrate, 0.1%Triton X-100 in water), the tissue slides were washed twice with PBS at room temperature. Non-specific binding sites were blocked by 1 h incubation with 2% bovine serum at room temperature in a humidified chamber, and the tissues were probed with rabbit anti-COX-1 or COX-2 (murine) primary antibody diluted in blocking serum 1:100 overnight at 4°C. The slides were washed twice with PBS, and the tissues were probed with Alexa Fluor 488-conjugated anti-rabbit secondary antibody diluted 1:200 (Cell Signaling Technology) for 1 h at room temperature in a humidified chamber protected from light. The slides were washed once with PBS and counterstained with Hoechst nuclear dye followed by two rinses with water. The tissues were covered with 0.1% para-phenylenediamine (PPDA) anti-fade reagent and observed under a fluorescence microscope. Negative controls were performed using the same protocol but substituting the primary antibody with rabbit anti-GST primary antibody (Cell Signaling Technology).

### Protein extraction and western analysis

Protein homogenates from the pregnant rat endometrium were isolated as described previously [12]. Briefly, uteri from day 2 to day 20 pregnant rats were rapidly excised and placed in ice-cold saline until dissected. Uteri were carefully laid on a glass plate and placed on the stage of a dissecting microscope. In early pregnancy (day 2 to 5.5), total endometrium was scraped using a microscope glass and collected. Uteri from day 6 to 10 the placenta and decidua were at an early stage of differentiation and could not be reliably separated. For this reason, decidua basalis (DB) dissected from animals between these days of pregnancy contains some chorioallantoic cells, but antimesometrial decidua, choriovitelline tissues, fetus, and myometrium were removed. Even though we carefully dissected DB from these tissues, it is a possibility that a contamination with some antimesometrial decidual cells that regress to form the deciduas caspularis (DC) would occur. In uteri collected from Day 12 to 20 pregnant rats, DB was isolated by gently separating the placenta and myometrial regions with 23-gauge needles. Additionally, the DB began to regress on Day 14 and became too thin to reliably dissect after Day 17. The protocol for DB isolation was described previously by Ogle and George [13].

Endometrial cells from pregnant animals were homogenized using a pipette in RIPA lysis buffer (PBS 1× pH 7.4; 1% Nonidet P-40; 0.5% Sodium deoxycholate; 0.1% SDS; Protease Inhibitor Cocktail Tablets (Roche Diagnostics Canada, PQ)). Homogenates were centrifuged (12,000 × g for 20 min at 4°C) to remove insoluble material. The supernatant was recovered and stored at -20°C till further analysis. Protein content was determined with the Bio-Rad DC Protein Assay. Protein extracts (50 μg) were heated at 94°C for 3 min, resolved by 10% SDS-PAGE and electrotransferred to nitrocellulose membranes using a semidry transfer apparatus (Bio-Rad, Mississauga, ON). The membranes were then blocked for 2 hrs at room temperature with PBS containing 5% milk powder, then incubated with anti COX-1 1:1000 and COX-2 1:2000 and subsequently with horseradish peroxidase-conjugated anti-rabbit secondary antibody (1:3000; room temperature for 45 min). All membranes were stripped with Restore™ western blot stripping buffer (Pierce, # 21059, lot # FH71541), and reprobed with an antibody specific to β-actin which was used as an internal standard. Peroxidase activity was visualized with the Super signal^® ^West Femto maximum sensitivity substrate (Pierce, Arlington Heights, IL, USA), according to the manufacturer's instructions. Signal was visualized using the Biochemi Imaging System (UVP, CA, USA). Densitometrical analyses were performed (protein of interest and β-actin) using the GelDoc 2000 and the Quantity One software (Bio-Rad, Mississauga, ON, Canada). Results were expressed as a ratio (protein of interest/β-actin) to correct for loading for each endometrial sample.

### Prostaglandin metabolites enzyme immunoassay

After collection from the animals, blood was allowed to clot for 30-60 min at 37°C. The serum was then separated from the clot and any remaining insoluble material removed by centrifugation at 3500 rpm for 10 min at 4°C. After preparation, serums were kept at -80°C until further use. EIAs for prostaglandins metabolites were performed according to manufacturer's instructions. Briefly, 50-μl of serum was used for each of the PGF2α and PGE2 metabolites in 96-well plates coated with goat anti-rabbit IgG antibodies. 50 μl of metabolite tracer and 50 μl of the metabolite specific antibody were added to each well. The plates were incubated overnight at 4°C or room temperature depending on the metabolite. Then, wells were washed five times with 10 mM phosphate buffer (pH 7.4) containing Tween-20 (0.05%) at pH 7.4; and 200 μl of Ellman's reagent (69 mM acetylthiocholine and 54 mM 5,5'-dithio-bis [2-nitrobenzoic acid] in 10 mM phosphate buffer, pH 7.4) was added to each well and plates were incubated in the dark at room temperature for 60-90 min. This allowed the bound enzyme tracer to react with Ellman's reagent to yield a yellow solution that can be measured photometrically with a microplate reader at 420 nm. A standard curve was made simultaneously with standards of the PGs metabolites, and determination of metabolite concentrations relative to those standards was calculated.

### Statistical analysis

All the experiments of pregnant animals were repeated 3-4 times. Endometrial extracts from each rat were assessed individually. Results subjected to statistical analyses were expressed as mean ± SEM. Data were subjected to one-way ANOVA (PRISM software version 4.0; GraphPad, San Diego, CA). Differences between experimental groups were determined by the Tukey's test.

## Results

### Expression of COX-1 protein in the pregnant rat endometrium

**Figure 1:**
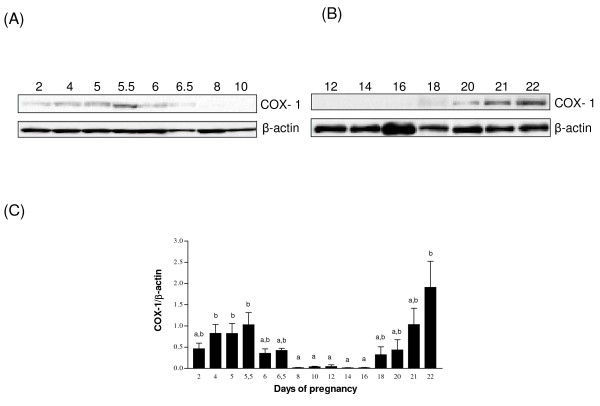
**The expression of COX-1 protein in rat endometrium during pregnancy**. Total endometrial proteins were collected at indicated days of pregnancy and were analysed by western blotting. Representative blots are shown (1A&1B). Histograms show densitometric analysis of COX-1 normalized to β-actin to correct for protein loading (1C). Data represents the mean ± SEM of 3-4 independent experiments. Columns with different superscripts are significantly different (p < 0.05).

Western analyses were performed using lysates of pregnant uteri to analyze COX-1 protein levels during rat pregnancy (Fig. 1). We found that COX-1 protein was significantly increased at days 4 to 5.5 of early pregnancy (implantation) and its expression was maximal at days 21 and 22 (parturition). Moreover, the enzyme was almost undetectable from days 8 to 16 of pregnancy (Fig. 1).

### Expression of COX-2 in the pregnant rat endometrium

**Figure 2:**
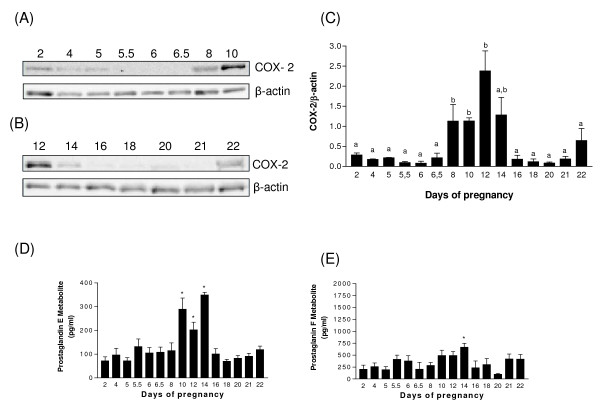
**The expression of COX-2 protein in rat endometrium during pregnancy**. Total endometrial proteins were collected at indicated days of pregnancy and were analysed by western blotting. Representative blots are shown (2A & 2B). Histograms show densitometric analysis of COX-2 normalized to β-actin to correct for protein loading (2C). Data represents the mean ± SEM of 3-4 independent experiments. Columns with different superscripts are significantly different (p < 0.05). Concentration of PGE2 and PGF2 α metabolites in the serum of pregnant rats (2D&2E). Serum samples were collected at indicated days of pregnancy and the metabolites were measured using a high sensitivity enzyme immunoassay (EIA) kit. Data represents the mean ± SEM of four independent experiments. *Significantly different from all other days of pregnancy (p < 0.05).

To document the trends of COX-2 protein in the rat uterus during pregnancy, western analyses were performed on lysates of pregnant rat endometrium (Fig. 2). Our results showed that COX-2 protein was not present at all days of pregnancy but was rather induced during specific stages of pregnancy. COX-2 protein was present in pregnant rats endometrial cells from days 2 to 5 (pre-implantation period) and was induced from days 8 to 12; maximal level was reached at day 12 (decidual regression). The enzyme was undetectable from days 18 to 21 but was induced at days 21 and 22 (parturition).

To determine if the augmentation of cyclooxygenases at specific times of pregnancy was followed by an increase in production of PGE2 and PGF2α, PGE2 (13,14-dihydro-15-keto metabolite) and PGF2α (13,14-dihydro-15-keto-PGF2α) metabolites were measured at the same time by EIA in serum from pregnant rats. According to our results, PGE2 and PGF2α metabolites were slightly increased at day 5.5 of pregnancy as compared to day 1-5 (Fig. 2D and 2E). Statistically significant increase in levels of prostaglandin E2 and F2α metabolites was observed at days 10, 12 and 14 for PGE2 metabolite (Fig. 2D), and at day 14 for PGF2α metabolite (Fig. 2E) during decidua basalis regression.

### Expression of COX-1 and COX-2 during pseudopregnancy

**Figure 3:**
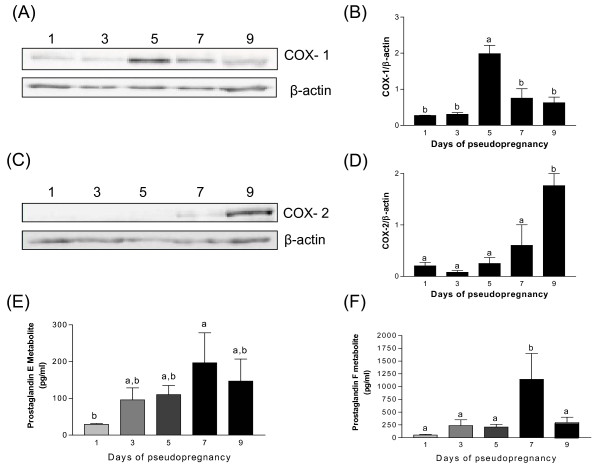
**The expression of COX-1 (3A) and COX-2 proteins (3C) in rat endometrium during pseudopregnancy**. Total endometrial proteins were collected at indicated days of pseudopregnancy and were analysed by western blotting. Representative blots are shown. Histograms show densitometric analysis of COX-1 and COX-2 normalized to β-actin to correct for protein loading (3B&3D). Data represents the mean ± SEM of 3-4 four independent experiments. Concentration of PGE2 and PGF2α metabolites in serum from pseudopregnant rat (3E&3F). Serum samples were collected at indicated days of peudopregnancy and the metabolites were measured using a high sensitivity enzyme immunoassay (EIA) kit. Data represents the mean ± SEM of four independent experiments. Columns with different superscripts are significantly different (p < 0.05).

To determine whether COX proteins were modulated by 17β-estradiol and progesterone in the endometrium during pregnancy, Western analyses were performed on lysates from a model of pseudopregnant rat endometrium (Fig. 3A-D). COX-1 was significantly increased at day 5 of pseudopregnancy (decidualization), while COX-2 was undetectable at day 5 but increased gradually and was maximal at day 9 (period preceding decidual regression). These results indicate that sex steroids can modulate the expression of COX enzymes in the endometrium, although the two enzymes would be differentially regulated. During pseudopregnancy, PGE2 and PGF2α metabolites similarly reached maximal concentration on day 7 (Fig. 3E and 3F).

### COX-1 and COX-2 expression in female rat endometrium during the estrous cycle

**Figure 4:**
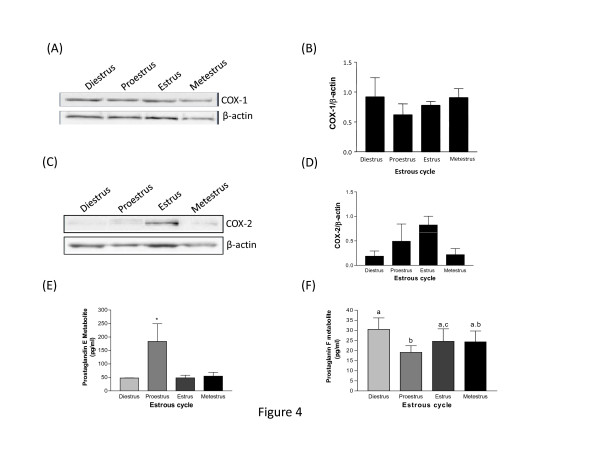
**The expression of COX-1 (4A) and COX-2 proteins (4C) in rat endometrium during estrous cycle**. Total endometrial proteins were collected at indicated days from polyoestrus cycling rats at each stage of the estrous cycle (diestrus, proestrus, estrus and metestrus) and subjected to western blots analysis of COX-1 and COX-2 content. Representative blots are shown. Histograms show densitometric analysis of COX-1 and COX-2 normalized to β-actin to correct for protein loading (4B&4D). Data represents the mean ± SEM of 3-4 independent experiments. Concentration of PGE2 and PGF2 α metabolites in serum of polyoestrus cycling rats at different stages of the estrous cycle (diestrus, proestrus, estrus and metestrus) (4E&4F). Serum samples were collected at indicated stage of estrous cycle and the metabolites were measured using a high sensitivity enzyme immunoassay (EIA) kit. Data represents the mean ± SEM of four independent experiments. Columns with different superscripts are significantly different (p < 0.05).

Western analyses performed on endometrial lysates at different phases of the estrous cycle (diestrus, proestrus, estrus and metestrus) revealed a higher level of COX-2 at the estrus phase compared to the other phases (Fig. 4C). There was no significant change in COX-1 protein expression (Fig. 4A). However, during the estrous cycle, significantly higher levels of PGE2 and PGF2α metabolites were observed at proestrus phase (Fig. 4E and 4F).

### Localization of COX-1 and COX-2 proteins in rat endometrium

**Figure 5:**
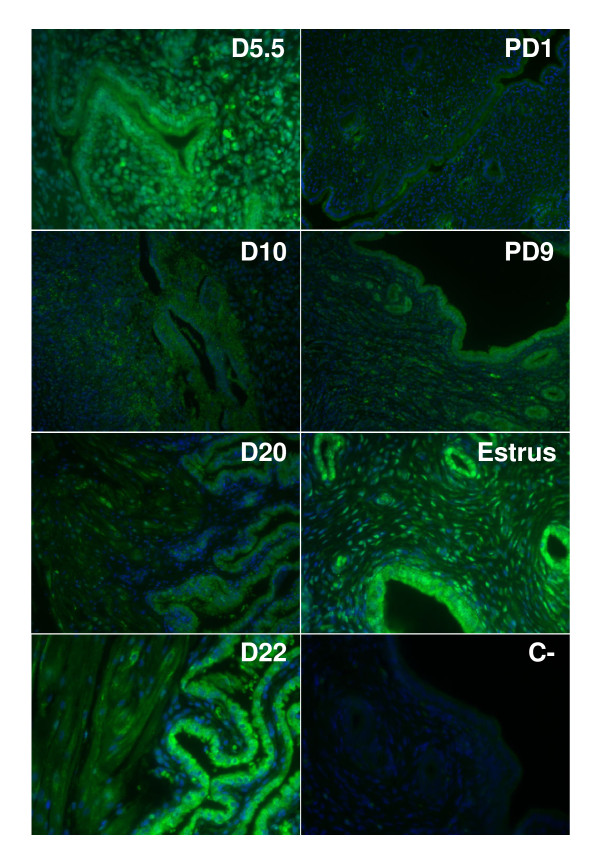
**Immunofluorescence staining of COX-1 protein in rat endometrium during pregnancy, pseudopregnancy and estrous cycle**. COX-1 localization (green color) was assessed in rat endometrium during pregnancy (days 5.5, 10, 20 and 22), pseudopregnancy (days 1 and 9) and estrous cycle (at estrus). Tissues were counterstained with Hoechst to stain nuclei (blue). For negative control (C-) primary antibody was replaced by normal rabbit IgG. Results are representative of three independent experiments. Magnification: 200×. PD: pseudopregnancy.

**Figure 6:**
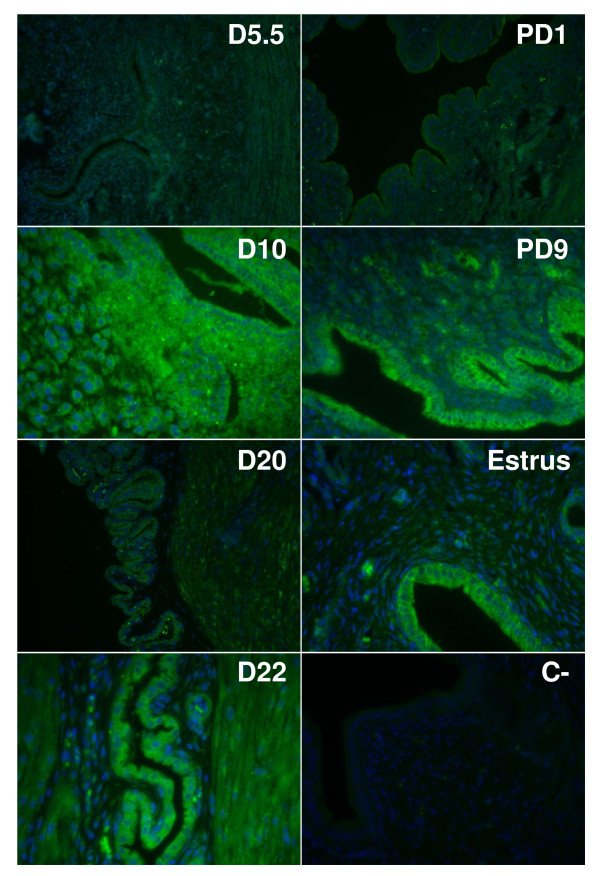
**Immunofluorescence staining of COX-2 protein in rat endometrium during pregnancy, pseudopregnancy and estrous cycle**. COX-2 localization (green color) was assessed in rat endometrium during pregnancy (days 5.5, 10, 20 and 22), pseudopregnancy (days 1 and 9) and estrous cycle (at estrus). Tissues were counterstained with Hoechst to stain nuclei (blue). For negative control (C-) primary antibody was replaced by normal rabbit IgG. Results are representative of three independent experiments. Magnification: 200×. PD: pseudopregnancy.

To localize COX-1 and COX-2 proteins, uterine tissues from pregnant rats were sectioned and immunofluorescence analyses were performed (Fig. 5 and 6). The results showed that both enzymes were mostly present in luminal and glandular endometrial epithelial cells, but also to a lesser extent in the stroma. We have found high levels of COX-1 immunoreactivity on days 5.5 (implantation) and 22 (parturition) and at day 9 of pseudopregnancy (Fig. 5). As observed using western analyses, COX-1 expression was low on day 10 of pregnancy and day 1 of pseudopregnancy. COX-2 signal at estrus, and on days 10 and 22 of pregnancy were found to be maximum (Fig. 6). Expression of COX-2 was low on days 5.5 and 20 of pregnancy. These results were in accordance with COX-1 and COX-2 protein expression as demonstrated by western analyses (Fig. 1, 2, 3, 4).

### *In vitro *modulation of cyclooxygenases in primary stromal cells

**Figure 7:**
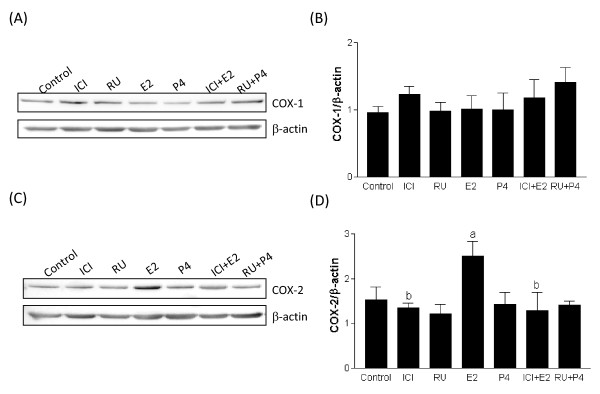
**The expression of COX-1 (7A) and COX-2 proteins (7C) in primary cultured rat stromal cells**. The stromal cells were pre-treated with anti-estrogen ICI-182,780 and anti-progestin RU-486 at concentration of 1 μM for 1 hr, followed by stimulation with estradiol and progesterone at concentration of 100 nM for 48 h. Histograms show densitometric analysis of COX-1 and COX-2 normalized to β-actin to correct for protein loading (7B&7D). Data represents the mean ± SEM of four independent experiments. Columns with different superscripts are significantly different (p < 0.05).

In order to study and confirm the role of steroid hormones in modulation of COX-1 and COX-2, primary stromal decidual cells from rat uterus were treated with estradiol and progesterone in the presence and absence of their respective antagonists (Fig 7). Our results showed that there was no significant change in the protein expression of COX-1(7A&7B), whereas COX-2 levels were found to be increased in the presence of estradiol (Fig 7C&7D). Indeed, pre-treatment of stromal cells with ICI-182,780 prevents the increase in COX-2 with estradiol treatment. In contrast, progesterone and its antagonist RU-486 had no effect on the modulation of cyclooxygenases.

## Discussion

In the last few years our laboratory has developed an expertise in female rat reproductive functions [7,14,15]. In the present study, we have used rat as a model to characterize the presence, activity and regulation of COX-1 and COX-2 by sex steroids at specific stages in the pregnant, pseudopregnant and non-pregnant rat uterus.

COX-1 is known to be a constitutive enzyme that plays "housekeeping" functions in many tissues. Our results, nonetheless, demonstrate that COX-1 protein level can be down-regulated in the rat endometrium; indeed, COX-1 expression was almost undetectable from days 10 to 16 of pregnancy during decidual regression period. COX-1 expression is probably regulated by sex steroid hormones *in vivo*, since COX-1 levels were elevated at specific days of pseudopregnancy. The increase in COX-1 expression at the time of parturition is in agreement with the studies by Reese and collaborators, who had shown that COX-1 is a major source of PGs during parturition and is essential for successful parturition [3]. Collectively our results suggest the possible involvement of COX-1 in implantation and parturition, but not in the process of decidual regression.

In this study, we did not observe a significant increase of COX-2 during implantation, but COX-2 has also been detected at the beginning of pregnancy from day 2 to 5. Increase in COX-2 expression in epithelial and stromal cells was also detected using immunostaining which is in accordance with the study by Cong *et al *[16]. In the past, gene knockout studies have revealed that COX-2 -/- mice showed implantation and decidualisation defects during pregnancy [4,5]. Interestingly, experiments with COX-1 -/- mice revealed that the loss of COX-2 can be compensated by COX-1 to rescue female infertility [17]. By analyzing the quantity of PGE2 and PGF2α metabolites during pregnancy and pseudopregnancy, we observed an augmentation of both metabolites at from day 10-14 in case of pregnancy and day 7 of pseudopregnancy and the trends were similar to COX-2 protein expression. These results suggest that the augmentation of COX-2 is responsible in majority for the augmentation of PGE2 and PGF2α during this period. Therefore, COX-2 plays a major role during the regression of decidua by producing PGE2 and PGF2α. Moreover, the variation of COX-2 expression during pseudopregnancy indicates that the enzyme is not only influenced by the implanting blastocyst during early pregnancy but also by steroid hormones produced by maternal tissue. This was further confirmed by *in vitro *stromal cell culture, where COX-2 expression was found to be modulated by estradiol treatment. Moreover, in normal pregnancy COX-1 is increased through day 5.5 and COX-2 is gradually increased from day 8 to 12, while in the pseudopregnant model, COX-1 expression is maximal at day 5 and COX-2 expression is maximal at day 12. Thus, these results further suggest that the model of pseudopregnancy mimics well the *in vivo *situation.

During estrous cycle, our results demonstrated a significant increase in PGE2 metabolite at proestrus phase where apoptotic death is known to be absent. Recent studies showed that PGE2 induces proliferation of human endometrial epithelial cells [18]. The significant increase of PGE2 metabolite at proestrus may suggest its implication in cellular proliferation as seen in the endometrium during this period preceding embryo implantation. It is clearly evident from the studies of Kennedy et al.[19] that PGE2 is involved in decidualization. In the present study, we also demonstrated that PGE2 might be involved in decidua basalis regression since it is significantly increased during this specific period of pregnancy. Recent study from our laboratory has shown that TGF-β isoforms are increased in the decidua basalis and induced apoptosis in decidual cells *in vitro *suggesting that TGF-β might be involved in decidua basalis regression [15]. Whether TGF-β induces decidual cells apoptosis through the induction of COX-2 and consequently an increase of prostaglandins remains to be elucidated during rat pregnancy. Some studies have suggested a role for TGFβ1 in the induction of COX-2 and PGE2 production in mink lung epithelial cells (Mv1Lu) [20]. Thus similar mechanisms could be involved in the regulation decidua basalis regression in the rat endometrium during pregnancy.

In conclusion, these results characterize the expression and localization of COX-1 and COX-2 in the non-pregnant, pregnant and pseudopregnant rat uterus and also show that these two enzymes are the key factors involved in embryo implantation, decidualization, decidua basalis regression and parturition. Further, the presence of these enzymes in the endometrium might be directly or indirectly involved in the control of cell apoptosis during implantation and decidual regression which could be regulated by steroid hormones.

## Competing interests

The authors declare that they have no competing interests.

## Authors' contributions

ISL carried out experiments, performed data analysis, and drafted the manuscript; MS performed *in vitro *studies and reviewed the manuscript, KB, SP and VL participated in the *in vivo *experiments; EA conceived and designed the study and finalized writing of the manuscript. All authors read and approved the final manuscript.
